# Trimerization of the N-Terminal Tail of Zika Virus NS4A Protein: A Potential In Vitro Antiviral Screening Assay

**DOI:** 10.3390/membranes11050335

**Published:** 2021-04-30

**Authors:** Janet To, Jaume Torres

**Affiliations:** School of Biological Sciences, Nanyang Technological University, 60 Nanyang Drive, Singapore 637551, Singapore; janetto@ntu.edu.sg

**Keywords:** Zika virus, NS4A, oligomerization, liposomes, amphipathic helices

## Abstract

The nonstructural (NS) protein NS4A in flaviviruses is a membrane protein that is critical for virulence, and, among other roles, it participates in membrane morphogenesis. In dengue virus (DENV), the NS4A hydrophilic N–terminal tail, together with the first transmembrane domain, is involved in both homo-oligomerization and hetero–oligomerization with NS4B. In both DENV and Zika virus (ZIKV), this N-terminal tail (residues 1–48) forms a random coil in solution but becomes mostly α-helical upon interaction with detergents or lipid membranes. Herein, we show that a peptide from ZIKV NS4A that spans residues 4–58, which includes most of the N–terminal tail and a third of its first transmembrane domain, forms homotrimers in the absence of detergents or liposomes. After interaction with the latter, α–helical content increases, consistent with binding. The oligomeric size of NS4A is not known, as it has only been reported in SDS gels. Therefore, we propose that full-length NS4A forms homotrimers mediated by this region, and that disruption of the oligomerization of peptide ZIKV NS4A 4–58 in solution can potentially constitute the basis for an in vitro assay to discover antivirals.

## 1. Introduction

Zika virus (ZIKV) was first isolated in the Zika Forest of Uganda almost 70 years ago [1] in the serum of a monkey. Transmission to the Americas resulted in the infection of more than a million people in 2015 [2]. Since 2015, Zika has been spreading with alarming rapidity, with outbreaks reported in 87 countries [3,4]. ZIKV is a member of the *Flavivirus* genus, within the *Flaviviridae* family. This family also includes important human pathogens, e.g., hepatitis C virus (HCV), yellow fever virus (YFV), West Nile virus (WNV), and dengue fever virus (DENV) [5]. *Aedes* mosquitoes are the vectors of the natural transmission cycle [6,7,8], but sporadic reports exist of direct human-to-human transmission [9,10,11].

An estimated 80% of ZIKV-infected people are asymptomatic, and the rest experience just an influenza-like syndrome [12]. However, thousands of cases of microcephaly and other neurological disorders have been recorded [13,14,15,16,17], whereas changes in gene expression occur during TLR3 activation and involve over 40 genes that disrupt neurogenesis [18]. Indeed, ZIKV targets human brain cells, reducing their viability and growth [19]. ZIKV infection has also been associated with Guillain–Barre syndrome (GBS), an autoimmune disease causing acute or subacute flaccid paralysis that can even cause death [20], although this link has not been confirmed [21].

There are no vaccines or antiviral agents available to treat ZIKV infection [3,22], and treatment with analgesics and antipyretics is directed to relieve symptoms [23]. Therefore, developing vaccines and antivirals against ZIKV is a current relevant challenge.

Members of the *Flavivirus* genus have a single-stranded positive–sense RNA genome [24]. This encodes a long polyprotein that is eventually cleaved by proteases into the structural proteins capsid (C), premembrane/membrane (prM/M), and envelope (E). In addition, non-structural (NS) proteins crucial for replication are NS1, NS2A, NS2B, NS3, NS4A, NS4B, and NS5. Entrance to the cell [8] is followed by internalization in endosomes, and low pH triggers the fusion of the viral envelope and the endosomal membranes [25]. The C protein associates with genomic RNA to form the virion core, whereas prM assists E protein folding while preventing its premature fusion [26]. Enveloped immature virions bud into the endoplasmic reticulum (ER) and are trafficked through the Golgi complex [25,26]. RNA synthesis is performed in the replication complex (RC), a virus-induced membrane network derived from the ER [27,28,29] that contains non-structural proteins, viral RNA, and host cell factors [30,31,32].

**Figure 1:**
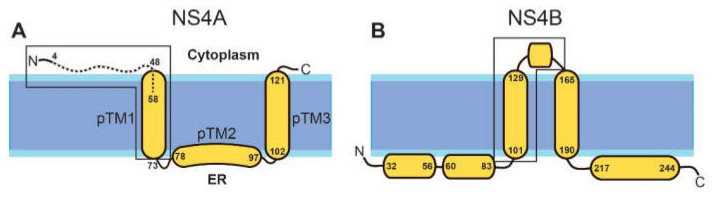
Schematic representation of NS4A and NS4B in DENV. The topology and helical domains of the corresponding proteins in ZIKV are assumed to be the same. (**A**) NS4A: the N-terminal peptide (4–58) used in the present work is shown as a broken line. Previous works used the peptide 1–48 (see main text). The N-terminal domain (first 60 residues) of NS4A proteins in ZIKV (MR–766) and dengue 1 and 2 has a high similarity (37% identity, 66% similarity). The approximate domain responsible for homo-oligomerization and hetero-oligomerization with NS4B is inside a box; (**B**) NS4B: beginning and end of α-helices indicated. The region involved in hetero-oligomerization with NS4A is inside a box.

In DENV, where NS4A has been studied in detail, it has been reported to be sufficient to induce membrane alterations that resemble the highly curved membranes typical of RCs [31]. The topology and secondary structure of the mature protein (Figure 1A) have a water soluble N-terminal cytoplasmic domain (residues 1–48), followed by three predicted transmembrane (pTM) segments [33], although only pTM1 and pTM3 span the membrane [31,34]. The N-terminal peptide (1–48) is linked to cytopathic effects [35].

When isolated, the N-terminal tail (1–48) forms a random coil in solution but becomes α-helical upon interaction with detergent or membranes [36,37,38,39]. Full-length NS4A forms homo-oligomers mediated by both the N-terminal cytoplasmic region [36] and pTM1 (residues 50–76) [40], but the size of the homo-oligomer is unclear. In one study [40], DENV NS4A oligomeric size was studied in SDS gels, using either protein expressed in infected cells or recombinant purified protein, but results were not conclusive; the protein expressed in infected Vero and BHK–21 cells formed monomers and dimers, whereas several oligomers, up to tetramers, were observed with the purified protein. In another study [34], purified NS4A was crosslinked, but an SDS gel only produced monomers and some dimers.

In DENV, NS4A also forms hetero-oligomers with NS4B, which, in its mature form, has five major helical domains: two reside in the ER lumen and three are transmembrane, although the last one is cleaved and translocates to the ER luminal side (Figure 1B). These hetero-oligomers involve residues 40–76 in NS4A [41] (Figure 1A), and one TM domain and a cytoplasmic loop in NS4A (Figure 1B, see box). Therefore, there is a clear overlap in the regions involved in homo– and hetero–oligomerization of NS4A, as they both involve the N-terminal tail and the first TM domain. NS4A homo- and hetero-meric interactions are critical for replication [40] and for temporal/spatial regulation during the viral infection cycle. Therefore, their disruption is a potential avenue for the discovery of antivirals [42,43,44].

The structure and functions of ZIKV proteins are likely to be similar to those found in other flaviviruses, e.g., DENV [45,46,47]. In fact, NS4A in DENV and ZIKV have the same length (127 residues) and probably identical topology. The three pTMs, according to the TMHMM prediction [48], involve similar residues to those of DENV NS4A: 51–73, 78–97, and 102–121 (Figure 1A). Its N-terminal cytoplasmic tail (residues 1–48) also has a random coil structure in solution and, like that of DENV, it becomes ~50% α-helical in the presence of detergent or lipid membranes [36,37,38,39].

In the present study, we expressed both N-terminal cytoplasmic tail and full-length ZIKV NS4A in *E. coli* in order to obtain sufficient protein for future structural studies and to test the potential membrane morphogenic properties of its N-terminal tail. Interestingly, after screening a series of possible constructs, a truncated N-terminus (4–58) that extends up to a third of the first predicted transmembrane domain (Figure 1A) not only could be efficiently purified with just a His-tag but also produced homotrimers in the absence of detergent or liposomes.

## 2. Materials and Methods

### 2.1. Cloning of ZIKV NS4A and Expression

The nucleotide sequence of ZIKV NS4A protein (strain MR-766) was obtained from the National Center for Biotechnology Information (NCBI, Bethesda, Baltimore, MD, USA). The gene was synthesized and cloned into the expression vector pNIC28–Bsa4 for recombinant expression. More than 10 constructs, preceded by a TEV protease cleavage site and an N-terminal hexahistidine affinity tag, were tested in the NTU Protein Production Platform (NTU PPP, Singapore, Singapore) for expression screening in BL21 (DE3) Rosetta T1R *E. coli* bacteria (Supplementary File S1). The constructs were designed to cover different lengths of the NS4A N-terminal extramembrane domain, the three predicted transmembrane domains, and the full-length protein. Only one construct (residues 4–58) and the full-length protein could be successfully expressed and extracted in the presence of detergents, while all other constructs failed to express significantly.

Both constructs were expressed in soluble form in *E. coli* BL21 (DE3) Rosetta T1R in Terrific broth (TB). Protein induction was performed at 1 mM IPTG for 18 h at 18 °C. Harvested cell pellets were resuspended in lysis buffer (20 mM Tris-HCl, 300 mM NaCl, 5 mM imidazole, 10% glycerol, 2 mM β-mercaptoethanol, 5 mM PMSF, pH 8) and lysed with a sonicator. The cell lysate was centrifuged at 15,000× *g* for 30 min, and the supernatant was collected and filtered through a 0.2 µm syringe filter before purification. The supernatant was incubated with Profinity™ Ni-charged IMAC resin (Bio–Rad, Singapore, Singapore) overnight at 4 °C for binding, followed by washing with 25 mM imidazole and elution in 300 mM imidazole. For the full-length NS4A, 4 mM n-decyl-β-D-maltoside (DM) was included in the elution buffer. Protein-containing fractions were pooled and concentrated by ultrafiltration at 4 °C using a Amicon centrifugal filter unit (Merck, Darmstadt, Germany). Protein was further purified by size exclusion chromatography using a Superdex 200 Increase 10/300 GL column (GE Healthcare, Uppsala, Sweden) on an ÄKTA system, with elution buffer containing 20 mM Tris-HCl and 300 mM NaCl, pH 8 (in presence of 4 mM DM for full-length NS4A).

### 2.2. Circular Dichroism

The secondary structure was determined using circular dichroism (CD) from 185 to 260 nm at 20 °C (Chirascan, Applied Photophysics, Leatherhead, UK), and CD spectra were analyzed by CDSSTR in DichroWeb (Whitmore and Wallace 2004).

### 2.3. Static Light Scattering (SLS)

SLS was measured using a Zetasizer Nano-ZS instrument (Malvern, Worcestershire, UK) at 25 °C following established protocols [49]. The molecular weight of protein in solution was determined by static light scattering (SLS) from the Debye plot of (KC/R_θ_) vs. protein concentration [49], which ranged from 0.05 to 1.2 mg/mL. Peptide samples were filtered through a 0.02 µm Whatman Anotop^®^ 10 syringe filter (GE Healthcare, Freiburg, Germany) before the measurements. The Rayleigh ratio was calibrated using toluene, as instructed by the manufacturer.

### 2.4. Gel Electrophoresis

The protein concentration of samples was measured using a NanoDrop™ 1000 spectrophotometer (Thermo Scientific, Wilmington, DE, USA). SDS-PAGE gels were run at 200 V for 50 min, using TGS (25 mM Tris, 192 mM glycine, 0.1% SDS, pH 8.3) as a running buffer. Gels were stained with Coomassie blue (Bio–Rad, Hercules, CA, USA), and destained using 30% methanol and 10% acetic acid for the visualization of protein bands.

Blue-native polyacrylamide gel electrophoresis (BN-PAGE) was performed as previously described [50]. Briefly, purified protein was incubated in sample buffer containing 750 mM aminocaproic acid, 50 mM Bis–Tris HCl pH 7.0, 0.5 mM EDTA, and several detergents at four times their CMC. Coomassie brilliant blue was added to the sample to a concentration of 0.35% (*w*/*v*) immediately before gel loading. Samples were loaded into a precast NativePAGE™ Novex™ 4–16% Bis–Tris protein gel (Life Technologies, Carlsbad, CA, USA), with an inner blue cathode buffer (15 mM Bis–Tris HCl, 50 mM Tricine, and 0.02% Coomassie blue, pH 7.0) and an outer anode buffer (50 mM Bis–Tris HCl pH 7.0), and separated at 150 V for approximately 70 min at 4 °C. The blue cathode buffer was replaced with colorless cathode buffer (15 mM Bis–Tris HCl and 50 mM Tricine, pH 7.0) and allowed to run at 250 V till the dye front reached the edge of the gel. NativeMark™ (Life Technologies, Carlsbad, CA, USA) was used as molecular weight markers.

### 2.5. Liposome Preparation

Liposomes were prepared using the film rehydration method [51]. Briefly, a thin lipid film was formed by first dissolving the required amount of lipid powder (Avanti Polar Lipids Inc., Alabaster, AL, USA) in chloroform, followed by drying under a nitrogen gas stream. The film was kept in a vacuum desiccator for at least 2 h. POPC (1-palmitoyl-2-oleoyl-*sn*-glycerol-3-phosphocholine) or POPC/POPS (1-palmitoyl-2-oleoyl-sn-glycerol-3-phosphor-L-serine) liposomes of uniform size distribution were prepared by extrusion through 400 and 200 nm pore-size polycarbonate membranes using an Avestin extruder (Avestin Inc., Ottawa, Canada).

### 2.6. Liposome Aggregation Assay

The size of liposomes was determined by dynamic light scattering (DLS) using a Zetasizer Nano-ZS instrument (Malvern, Worcestershire, UK). Size variation was measured 0, 2, and 30 min after addition of the peptides to liposomes. Size changes elicited by ZIKV NS4A (4–58) and two predicted amphipathic α-helices (AHs), AH1 (15–33) and AH2 (38–55), were compared with those elicited by negative and positive control peptides, obtained from HCV NS4B N-terminal domain (AH1* (4–32) and AH2* (43–65)) [52].

### 2.7. Synthetic α-Helical Amphipathic Peptides

Peptides corresponding to two Zika NS4A, AH1 and AH2, and control peptides of HCV NS4B, AH1* and AH2*, (see above) were obtained by chemical synthesis (GenScript, Piscataway, NJ, USA) and purified by HPLC.

## 3. Results

### 3.1. Purification of N-Terminal Peptide (4–58) and Full Length (1–127) of ZIKV NS4A

**Figure 2:**
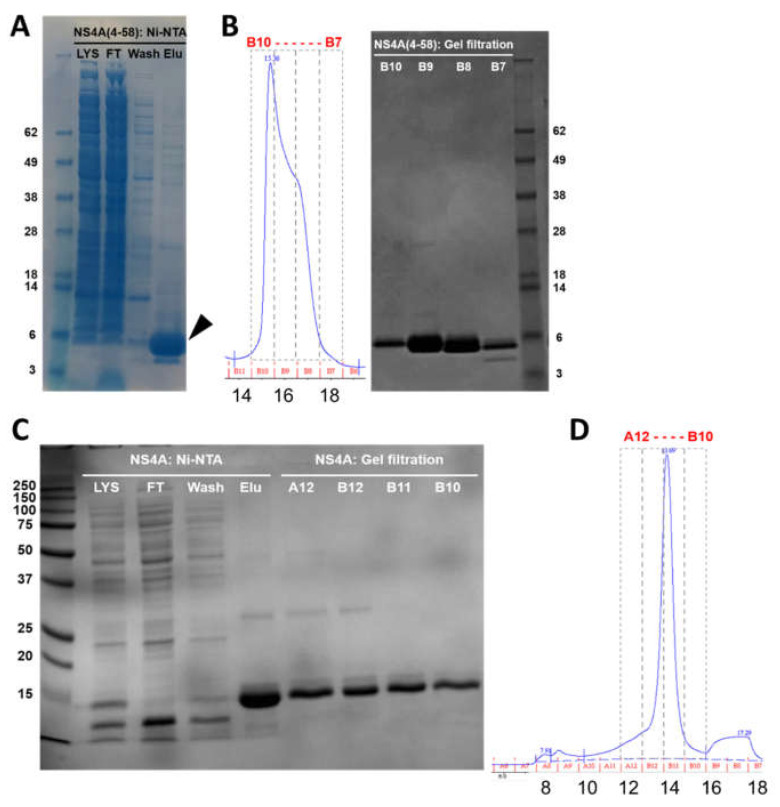
Purification of N-terminal tail (4–58) and full-length ZIKV NS4A protein. (**A**) SDS gel corresponding to elution fractions of peptide (4–58) after Ni-NTA chromatography; (**B**) left: SEC elution profile, right: SDS gel of fractions obtained from SEC; (**C**) SDS gel of the elution fractions of full-length NS4A after Ni-NTA chromatography (left) and after SEC (right); (**D**) SEC elution profile of full-length NS4A. Lys: lysate; FT: flow-through; wash: first wash; elu: elution. In SEC profiles, fraction numbers (top) and elution volumes (mL) (bottom) are indicated.

Both polypeptides were purified in two steps, first using Ni-NTA metal affinity chromatography, followed by size exclusion chromatography (SEC) (Superdex 200 Increase 10/300 GL column). The SDS-NuPAGE gel corresponding to the elution steps of NS4A (4–58) shows that the peptide was almost pure (Figure 2A, arrow). A single band can be observed that migrated slightly faster than its expected monomeric size (8.7 kD). This is not unexpected, as slower or faster migration in gels is characteristic of peptides that interact strongly with detergents or membranes, as is the case here (see below).

SEC showed that the peptide eluted at 15.4 mL, with a minor shoulder at 16.5 mL (Figure 2B, left). These two bands do not arise from impurities and must represent oligomers, since all the fractions were highly pure (Figure 2B, right). Indeed, the fraction at 15.4 mL (most intense) eluted between ovalbumin (44 kD) and carbonic anhydrase (29 kD), consistent with the formation of an oligomer, whereas the fraction at 16.5 mL may represent either a smaller oligomer or a monomer. The final yield of NS4A (4–58) was 8 mg/L of Terrific broth (TB) culture.

Full-length NS4A (expected molecular weight 16.5 kD) eluted in Ni-NTA affinity chromatography in the presence of 4 mM DM (n-decyl-β-D-maltoside), and it appeared pure (Figure 2C, Elu). After SEC, it eluted as a single peak at 13.7 mL (Figure 2D), consistent with a globular protein of ~70 kD. This volume also includes the detergent micelle, and it is also consistent with an oligomer, not with a monomer. All of the fractions obtained were pure (Figure 2C, lanes A12–B10). The yield for full-length NS4A was 35 mg/L of TB culture.

### 3.2. Blue-Native PAGE

**Figure 3:**
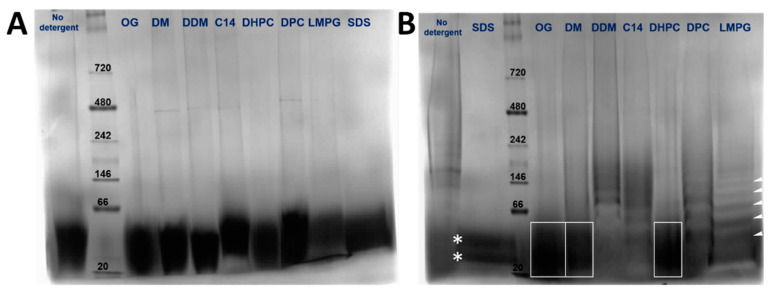
BN-PAGE of NS4A (4–58) (**A**) and full-length NS4A (**B**) in the detergents indicated above. In (**B**), some of the bands are highlighted to guide the eye (see text).

The oligomeric species present for the N-terminal tail (4–58) (8.7 kD) and full-length protein (16.5 kD) in various detergents was assessed using blue native polyacrylamide gel electrophoresis (BN-PAGE). Both in the absence of detergent and in all detergents screened, peptide (4–58) migrated as a single species between approximately 20–40 kD, consistent with a trimer or a tetramer (Figure 3A), although the gel was not resolved enough. In contrast, the results for the full-length protein were heavily dependent on the detergent used. The sample appeared more homogenous in OG, DM, and DHPC (Figure 3B, boxes). However, the bands were too poorly resolved to determine if more than one band was present, or their oligomeric size. In other detergents, there were obvious ladders of increasing molecular weight, e.g., in DDM, C14SB, DPC, and LMPG (see white arrows). In SDS, two species were observed in the range of 20–40 kD that could represent dimers and trimers (Figure 3B, stars). Overall, these data were not sufficient to determine the oligomeric size of peptide (4–58) or full-length NS4A.

### 3.3. Circular Dichroism Spectroscopy of Peptide NS4A (4–58)

**Figure 4:**
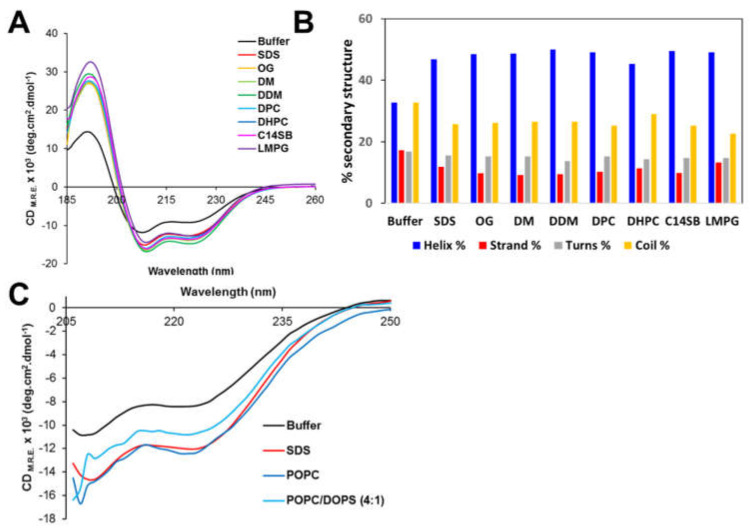
Circular dichroism spectra of peptide ZIKV NS4A (4–58). (**A**) CD spectra in aqueous solution (buffer) and in the presence of the indicated detergents; (**B**) percentage of secondary structure obtained in each condition in (**A**), according to DichroWeb fitting results (see Materials and Methods); (**C**) ellipticity after addition of liposomes of various compositions for wavelengths above 205 nm.

The secondary structure of peptide NS4A (4–58) in aqueous solution and in the presence of various membrane-mimicking detergent micelles was determined by far-UV CD. A peptide of ZIKV NS4A corresponding to the entire N-terminal cytoplasmic tail (residues 1–48) (see Figure 1A) forms a random coil in solution [37]. However, peptide (4–58) contained 33% α-helix in aqueous buffer (Figure 4A,B), and the addition of detergent increased α-helical content to 45–50% at the expense of β-structure and random coil. This clearly indicates that the peptide interacts with detergent micelles, as reported previously for peptide 1–48 [37]. In the presence of POPC liposomes, the α-helix content also increased similarly to detergent micelles (Figure 4C). This increase was lower in the presence of negatively charged membranes, POPC/DOPS. This suggests the peptide possesses less affinity for negatively charged membranes, as shown previously for its homolog peptide in DENV NS4A [36].

### 3.4. SLS of Peptide NS4A (4–58)

**Figure 5:**
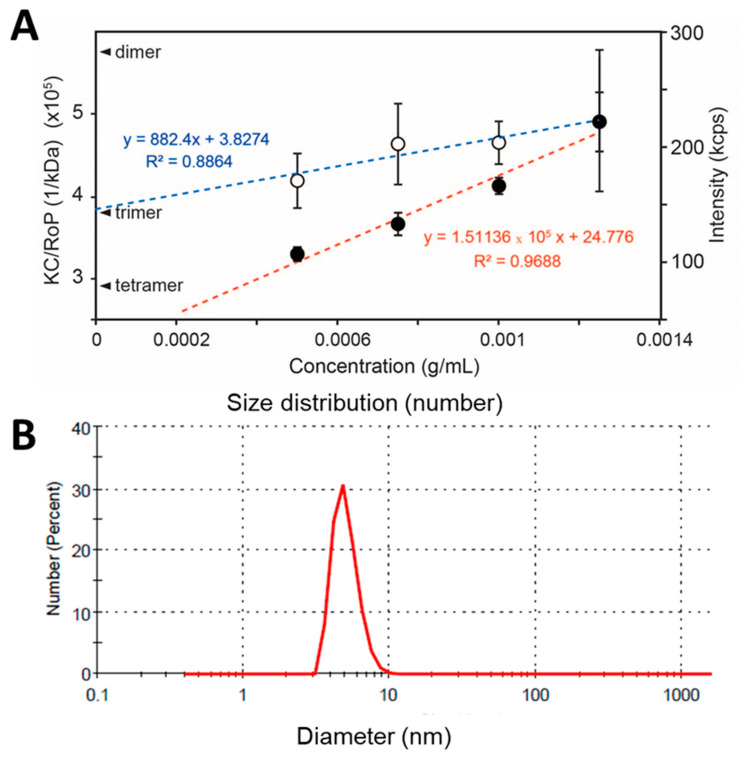
(**A**) Scattering properties of ZIKV NS4A (4–58) in aqueous solution. Scattering intensity (red line) and KC/RoP (blue line) plotted against protein concentration in mg/mL. The Y-intercepts corresponding to a dimer, trimer, and tetramer of an 8.7 kD monomer are indicated. The second virial coefficient, A_2_ (ml mol/g^2^) value obtained from the plot was 0.003262; (**B**) DLS of NS4A (4–58) in number representation in aqueous solution and in the absence of detergents or liposomes.

Since NS4A (4–58) is soluble in the absence of detergent, its oligomeric size was determined using static light scattering (SLS). Protein concentrations ranged from 0.5 to 1.5 mg/mL (n = 5 independent samples per concentration). The inverse of the y-intercept of the linear fit of KC/RoP produced a molecular weight of 25.0 ± 1.8 kD, clearly consistent with a trimer (Figure 5A). To confirm that the weight-averaged molecular weight obtained represents a monodisperse population and not an average of a heterogeneous mixture of sizes, the size distribution of the sample was also determined by DLS. This produced a single narrow band (in number representation) and no aggregates of larger sizes (Figure 5B). The average diameter was 4.9 ± 1.0 nm, consistent with the expected hydrodynamic size of a ~26 kD protein, as can be inferred from HydroPro [53] calculations of protein diameters obtained from the Protein Data Bank (PDB), for example, carbonic anhydrase I (PDB 2CAB: 29 kD, 4.9 nm), glutathione S-transferase (PDB 1B8X: 26 kDa, 4.8 nm), or STING monomer (PDB 4F5E: 28 kD, 4.9 nm). Thus, the molecular weight determined from SLS is consistent with a homotrimer of peptide 4–58.

### 3.5. Liposome Aggregation Assay

**Figure 6:**
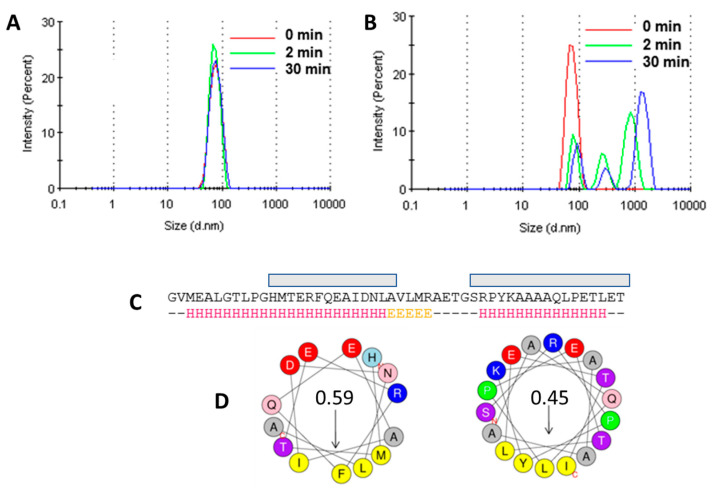
Liposome aggregation assay. (**A**) Effect of addition of AH1* to POPC liposomes (negative control); (**B**) same as (**A**) for peptide AH2* (positive control); (**C**) prediction of α-helical content in the N-terminal extramembrane domain of ZIKV NS4A (Jpred4, http://www.compbio.dundee.ac.uk/jpred/ accessed on 30 April 2021) and of highly amphipathic segments (gray boxes); (**D**) helical wheels showing amphipathicity values of these selected α-helical domains computed with Heliquest (http://heliquest.ipmc.cnrs.fr accessed on 30 April 2021). In this scale, hydrophobic moments ranging from 0.3 to 0.4 have significant amphipathic character [56], whereas hydrophobic moments >0.6 are considered highly amphipathic [57].

To test if NS4A (4–58) is able to aggregate liposomes, we conducted a liposome aggregation assay. As a positive control, we used an amphipathic AH2* peptide from the N-terminal extramembrane domain of HCV NS4B protein, known to promote large-scale vesicle aggregation [52]. HCV NS4B is essential for the creation of the membranous web [54,55], and small molecule viral replication inhibitors can successfully target this activity [52]. As a negative control, we used AH1* (see Materials and Methods), which does not promote vesicle aggregation. No change was observed for POPC liposomes alone, after DMSO addition, or after the addition of AH1* (Figure 6A). However, the addition of AH2* resulted in large aggregates (Figure 6B), as reported previously [52]. The addition of ZIKV NS4A peptide (4–58) did not produce any change in either DOPC or DOPC/DOPG liposomes (not shown).

Since one of the ways to modify membranes is by interaction with amphipathic α-helices, where hydrophobic sides of the latter insert in membranes, we also synthesized and purified two peptides corresponding to the ZIKV NS4A N-terminal tail, AH1 and AH2, predicted to have high amphipathicity when folded as α-helices (Figure 6C,D). The addition of these synthetic peptides did not produce any change in liposome size (not shown).

## 4. Discussion

Overall, we have shown that both a sequence of the ZIKV NS4A N-terminal domain (4–58) and the full-length protein can be easily purified without the need for fusion proteins, and in good yield. In contrast to previous reports that used the peptide 1–48 in NS4A of DENV and ZIKV [36,37], we found that the conformation of this peptide is not a random coil but is at least partially folded, even in the absence of detergents or lipid membranes. We hypothesize that the difference is due to the inclusion of residues that belong to one-third of the first TM domain of NS4A (49–58). We also found that peptide (4–58) binds membranes, since its conformation changes upon exposure to liposomes, in agreement with previous reports for both DENV and ZIKV NS4A [38,39]. However, neither peptide ZIKV NS4A (4–58) nor two putative amphipathic α-helical peptides, (15–33) or (38–55), produced any liposome aggregation activity in the conditions we used, in contrast to the positive control peptide AH2* from HCV NS4B.

It is interesting that this first TM domain, together with the cytoplasmic tail, has been previously reported to be involved in homo-oligomerization in DENV NS4A [36,40] and is strongly linked to cytopathic effects [35]. Our results support that this part of the protein is key for NS4A oligomerization. SEC results are consistent with both peptide (4–58) and full-length NS4A forming oligomers, although precise size could not be determined. Equally, results from BN-PAGE in different detergents were not conclusive. However, SLS results for peptide (4–58) show unequivocally that this peptide forms homotrimers in aqueous buffer in the absence of detergents or liposomes. In the literature, the oligomeric size of NS4A has been proposed to be a dimer, but this conclusion was only supported by bands in SDS gels, which often do not represent the behavior in milder detergents or in lipid membranes. Our results are consistent with ZIKV NS4A oligomerizing as trimers via the cytoplasmic tail and the N-terminal half of its first TM domain.

We propose that disrupting the oligomerization of this peptide may be targeted by antivirals. The N-terminal cytoplasmic tail of ZIKV NS4A is unlikely to be glycosylated, since it is exposed to the cytoplasm, and no glycosylation of NS4A has been reported experimentally [58]. Therefore, using a recombinant peptide obtained in *E. coli* like the one used here may form the basis of a useful in vitro screen assay. In DENV NS4B, mutations at the first TM domain have been reported to reduce virulence, and residues 84–146 have been shown to interact with residues 40–76 of the DENV NS4A protein [41] (Figure 1B). In NS4B, this part of the protein is also the target of drugs like NITD-618 (in DENV) [44,59], Dasatinib (in DENV) [60], CCG-4088 and CCG-3394 (in YFV) [61], or NITD-688 [62]. Drugs that destabilize the ZIKV NS4A (4–58) homotrimer could potentially disrupt NS4A–NS4B interactions.

## Data Availability

Not applicable.

## Supplementary Materials

The following are available online at https://www.mdpi.com/article/10.3390/membranes11050335/s1, Figure S1: Aqueous-soluble (A) and total (B) expression of ZIKV NS4A constructs preceded by a TEV protease cleavage site and an N-terminal hexahistidine affinity tag tested in the NTU Protein Production Platform (PPP, NTU Singapore) for expression screening in BL21(DE3) Rosetta T1R E. coli bacteria. Constructs were designed to cover different lengths of the NS4A N-terminal extramembrane domain, the three predicted transmembrane domains, and the full-length protein. Only one construct (residues 4–58) (A, green bar) and the full-length protein (B, green bar) could be successfully expressed and extracted in the presence of detergents, while all other constructs (red bars) failed to express significantly.
