# Supplementary material for: Trimerization of the N-Terminal Tail of Zika Virus NS4A Protein: A Potential In Vitro Antiviral Screening Assay
Source: Membranes (Basel). 2021 Apr 30;11(5):335. doi: 10.3390/membranes11050335 (PMC8147241; doi:10.3390/membranes11050335)
Supplement: Supplementary file 1 [file membranes-11-00335-s001.zip › membranes-1184443-supplementary.pdf]

membranes

MDPI

Supplementary Material

Trimerization of the N-terminal tail of Zika virus NS4A protein: a potential *in vitro* antiviral screening assay.

Janet To and Jaume Torres *

School of Biological Sciences, Nanyang Technological University, 60 Nanyang Drive, Singapore 637551; janetto@ntu.edu.sg
* Correspondence: jtorres@ntu.edu.sg

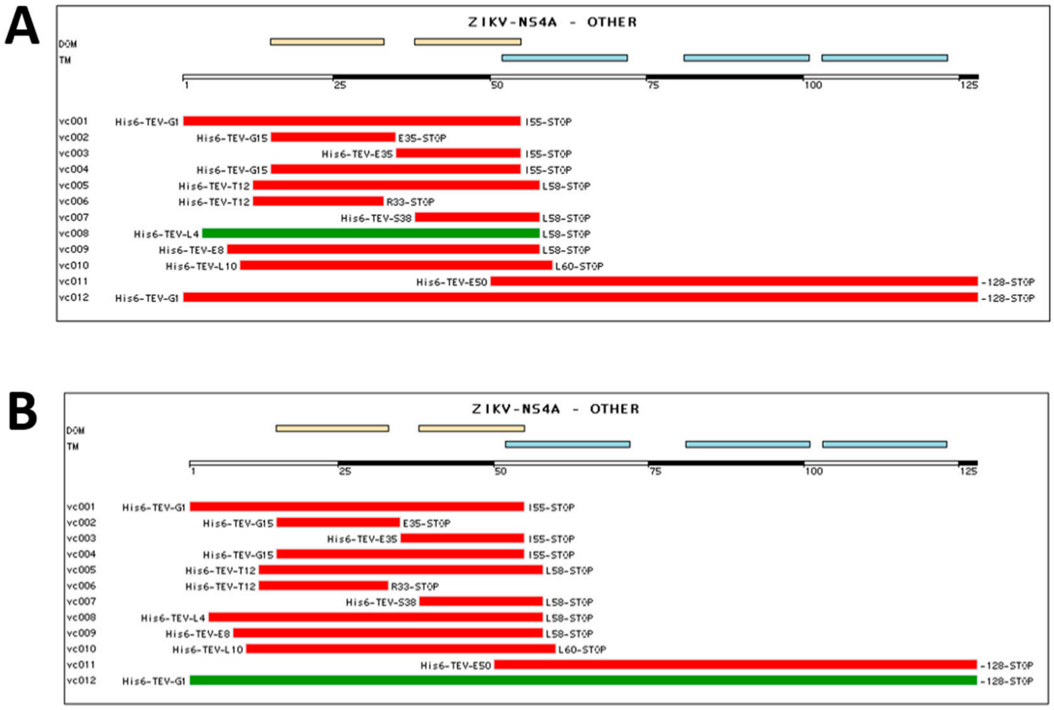

Figure S1. Aqueous-soluble (**A**) and total (**B**) expression of ZIKV NS4A constructs preceded by a TEV protease cleavage site and an N-terminal hexa-histidine affinity tag tested in the NTU Protein Production Platform (NTU PPP, Singapore) for expression screening in BL21(DE3) Rosetta T1R *E. coli* bacteria. Constructs were designed to cover different lengths of the NS4A N-terminal extramembrane domain, the three predicted transmembrane domains and the full-length protein. Only one construct (residues 4-58) (A, green bar) and the full-length protein (B, green bar) could be successfully expressed and extracted in the presence of detergents, while all other constructs (red bars) failed to express significantly.
